# Recorded Rates of Trauma‐Exposure in a Retrospective Epidemiologically Complete First‐Episode Psychosis Cohort

**DOI:** 10.1111/eip.13610

**Published:** 2024-08-31

**Authors:** Aisling Redmond, Keith Gaynor, Sean Naughton, Mary Clarke

**Affiliations:** ^1^ School of Psychology University College Dublin Belfield Dublin Ireland; ^2^ DETECT Early Intervention in Psychosis Service Blackrock Dublin Ireland; ^3^ School of Medicine University College Dublin Belfield Dublin Ireland

**Keywords:** FEP, psychosis, retrospective chart review, trauma, treatment planning

## Abstract

**Background:**

Trauma plays an important role in the development and maintenance of psychosis. However, it is still under‐examined in daily clinical practice. The current study investigated the rates of recording of trauma‐exposure in the clinical histories of a first‐episode psychosis (FEP) cohort attending an early intervention psychosis service.

**Methods:**

This study used a retrospective chart review methodology in a 6‐year epidemiologically complete FEP cohort attending an early intervention psychosis service. The Trauma and Life Events Checklist was used to define the rate and types of trauma‐exposure reported in clinical histories. The relationships were examined between recorded trauma‐exposure and positive and negative symptoms, depression and duration of untreated psychosis at first assessment.

**Results:**

Trauma‐exposure was frequently recorded within clinical histories. Childhood trauma‐exposure was recorded in 47.4% of the sample, which is lower than may be expected. No significant relationships between the recorded trauma‐exposure and symptom measures were found. A significant relationship was found between interpersonal stressors and positive symptoms, and work‐related stress and negative symptoms, highlighting the importance of proximal stressful life events.

**Discussion:**

This study found that clinicians were frequently recording trauma‐exposure in daily practice. However, it was unclear whether the recording of trauma‐exposure highlighted led to systematic diagnosis, assessment or treatment of trauma for people with psychosis. The importance of treatment and service planning to include information about trauma‐exposure is discussed.

## 1 Introduction

### 1.1 Research Evidence of the Trauma and Psychosis Relationship

Research has found a high level of trauma‐exposure in individuals who experience psychosis (Campodonico et al. 2021; Humphrey et al. 2022; Kilcommons and Morrison 2005; Panayi et al. 2022), leading to significant reported rates of PTSD, and C‐PTSD in people with psychosis (Bloomfield et al. 2021; Williams et al. 2018). A dose–response relationship between psychosis, PTSD, and trauma has been repeatedly found (Carr, Hardy, and Fornells‐Ambrojo 2018; Larkin and Read 2008; Matheson et al. 2013; Shevlin et al. 2008; Williams et al. 2018), leading the National Institute for Health and Care Excellence to recommend assessing individuals presenting with an FEP for PTSD and trauma‐exposure (National Institute of Clinical Excellence 2014).

The literature hypothesises that trauma is a key element of the biopsychosocial model of psychosis (Luhrmann et al. 2019). Childhood traumatic experiences are a risk factor for psychosis (Bentall et al. 2014; Varese et al. 2012; Vila‐Badia et al. 2021), with childhood interpersonal traumas being linked to the development and maintenance of psychotic symptoms (Bentall et al. 2014; Garety et al. 2001; Humphrey et al. 2022). Early life trauma may begin a chain of risk factors leading to a psychotic outcome. For instance, Kiburi et al. (2021) in their systematic review found that childhood trauma increased the risk of cannabis‐use and the subsequent development of psychosis. Individuals who have experienced trauma are more likely to display risky or challenging behaviours (Grattan et al. 2019), such as self‐harm (De Kloet et al. 2011), aggression (Ford et al. 2012), substance use (Ford and Fournier 2007), or homelessness (Grubaugh et al. 2011).

The type of trauma‐exposure appears to influence the type and content of psychosis presentations (Grattan et al. 2019; Hardy et al. 2016; Neill and Read 2022). A link between childhood sexual abuse and auditory hallucinations has been found. A study of 17 337 people found that self‐reported adverse childhood experiences doubled the risk of hallucinations (Whitfield et al. 2005) and childhood emotional abuse has been linked to delusions (Hardy et al. 2016). Delusions and hallucinations have been found to be related to negative evaluations of the self and others resulting from traumatic experiences (Kuipers et al. 2006).

Stressful life events which would not meet Criterion A for PTSD, are also a risk factor for developing psychosis (Collip et al. 2011), with workplace stress being found to increase the risk of developing psychosis (Amin 2023), and adulthood bullying has been linked to positive symptoms (O'Moore et al. 1998).

### 1.2 Trauma, Psychosis and Clinical Practice

Although populations experiencing psychosis have been found to have higher rates of childhood victimisation and PTSD than the general population (Conus et al. 2007; Kessler et al. 2005; Kraan et al. 2015; Steel, Doukani, and Hardy 2017), Read et al. (2018), in their systematic review, highlighted that a high proportion of clinical records contained no documentation of trauma in patients being treated for serious mental health problems. Nine studies of the 21 studies revealed that less than one‐third (28%) of abuse and neglect identified by researchers had been documented in clients' files, without any indication that it had been responded to therapeutically. There were even lower levels of recording for emotional neglect (17%) and physical neglect (10%). Similarly, Neill and Read (2022) investigating the documentation of trauma in community mental health services in England found that 87% of clinical records contained no documentation of adverse childhood experiences among patients. There is some evidence of slight improvement over time. Sampson and Read (2017) examined the extent to which abuse and neglect were identified and recorded by mental health services and compared it to a similar audit 20 years previously in the same service. There were significant increases, compared to the 1997 audit, in recorded rates of child sexual and physical abuse. However, identification of physical and emotional neglect was poor. People with a diagnosis of psychosis tended to be asked less often and had significantly lower rates of abuse/neglect identified. The disparity between the research evidence for the relationship between trauma and psychosis and clinical practice emphasises that a trauma‐focused approach had not become routine clinical practice. Staff may not inquire about trauma (Cavanagh, Read, and New 2004; Lab, Feigenbaum, and De Silva 2000; Mansfield et al. 2017; Young et al. 2001), patients may not disclose trauma (Cunningham et al. 2017), or the sequalae of potentially traumatic events gleaned in narrative histories may not be the subject of more detailed inquiry (Read et al. 2018).

A further consideration is that in recent years, many people may have had an increased risk of trauma‐exposure due to their experiences during the COVID‐19 pandemic (Delz et al. 2023; Kaubisch et al. 2022). Due to the recency of these events, it is unclear whether the COVID‐19 pandemic may have impacted on psychosis presentations in the community and the detection of this putative pathway may be hampered by limited systematic enquiry of trauma symptomatology in clinical histories.

### 1.3 Current Study

The current study examined a 6‐year dataset of intake assessments in an early intervention psychosis service to identify reported trauma‐exposure, characterise the reported traumas, and examine clinical correlates. The study hypothesised that there would be a low rate of trauma recorded, in line with the findings of Neill and Read (2022).

## 2 Material and Methods

### 2.1 Study Design

This study was designed as a retrospective chart review of a complete 6‐year cohort of FEP patients referred to an urban catchment area (adult population 150 000 approximately) within an early intervention psychosis service, DETECT. This a partial replication of Conus et al.'s (2007) retrospective analysis of trauma‐exposure in an FEP cohort attending EPPIC services in Melbourne, Australia. In the current study, every adult in contact with secondary mental health services within the geographically defined catchment area with a possible FEP is referred for assessment, diagnosis, and adjunctive interventions by the DETECT service. The thorough intake assessment includes an extensive history taking, SCID Interview, detailed symptom measures, and collateral interview.

### 2.2 Participants

All individuals who presented to the early intervention service with a FEP from 2017 to 2022 were included in the study. The sample size was *n* = 133, with an a priori power analysis indicating that appropriate statistical power was *n* = 107 (G*Power; Faul et al. 2009).

### 2.3 Materials

The Trauma Assessment and Life Experiences Checklist (TALE; Carr, Hardy, and Fornells‐Ambrojo 2018) defined exposure to a traumatic event. The TALE consists of 20 items relating to possible traumatic events an individual may have experienced. The TALE Interview was found to have satisfactory test–retest reliability convergent and construct validity (Carr, Hardy, and Fornells‐Ambrojo 2018). This was a novel use of Part A TALE as an assessment in a retrospective chart review methodology. Due to the ubiquity of experiences of psychotic symptoms and hospitalisation within the sample and without the opportunity to explore individual responses to these experiences through an interview, an alternative, the TALE 14 is reported here; these are the first 14 items of the TALE which indicate trauma‐exposure prior to the experience of psychosis. Exposure to trauma was dummy‐coded into binary variables. The age at which the trauma occurred was recorded, if reported in the assessment.

Work‐Related Stress (WRS) and Interpersonal Stressors were included as dependent dummy‐coded binary variables. WRS was defined as ‘*the response people may have when presented with work demands and pressures that are not matched to their knowledge and abilities, and which challenge their ability to cope*’ (Leka, Griffiths, and Cox 2003, 3). Interpersonal Stressors were defined as ‘*stressful episodes between two or more people that involve quarrels, arguments, negative attitudes or behaviour, an uncomfortable atmosphere during a conversation or activity, and concern about hurting others' feelings*’ (Kato 2013, 100).

Demographic variables collected were the age, sex, duration of untreated psychosis in months (DUP), service disengagement, and substance use were also collected. Service disengagement and substance use were binary‐coded. Sex was recorded as male or female in the case files, so no other options for sex are included in the recorded data.

The scales for the assessment of positive symptoms (SAPS) (Andreasen 1984a), and the scales for the assessment of negative symptoms (SANS) (Andreasen 1984b) were used to assess positive and negative symptom severity. The Calgary Depression Scale for Schizophrenia (CDSS) (Addington, Addington, and Schissel 1990) was used to assess depressive symptoms in psychosis. The Covid Traumatic Stress Symptoms scale from the Covid Stress Scales (Taylor et al. 2020) was used to identify retrospectively COVID‐19‐related trauma (CRT). The internal consistency, convergent and discriminant validity of the traumatic stress symptoms scale was satisfactory (Cronbach's *α* = 0.93) (Taylor et al. 2020).

### 2.4 Procedure

The current study was designed as a retrospective chart review. A pro‐forma data extraction form was developed based on demographic details, TALE items and the assessment questionnaires. The primary researcher reviewed each clinician‐completed FEP assessment, and extracted whether TALE items were recorded or not within the assessment report, including the case history and collateral interview. In line with the TALE instructions, a conservative approach to inclusion was utilised. Quantitative data from semi‐structured assessment scales and questionnaires was also extracted.

### 2.5 Data Analysis

Data were analysed using IBM's SPSS 27 software. Binary variables were dummy‐coded. TALE items were added to create a total score with possible scores ranging from 0 to 14 (TALE 14).

### 2.6 Ethics

The research ethics committees at University College Dublin and Saint John of God Community Services CLG approved this study. The study was conducted in line with the Declaration of Helsinki—Ethical Principles for Medical Research Involving Human Subjects (World Medical Association 2022).

## 3 Results

### 3.1 Missing Data Management

The data were screened for missing data. Five variables had missing data: DUP (14.3%), SAPS scores (3%), SANS scores (3%), CDSS scores (5.3%) and substance use (11.28%). This was managed through pairwise deletion (Peugh and Enders 2004). Little's MCAR test (*X* ^2^ = 17.15, *p* = 0.248) was used to confirm data was missing completely at random in line with the assumptions of pairwise exclusion.

### 3.2 Descriptive Statistics

The median age of the sample was 32 years. Fifty‐nine percent of the sample was male. Only 1.5% of the sample disengaged prior to assessment.

The data were assessed for normality. Due to a violation of the assumptions of normality, CRT, DUP, SANS and CDSS scores were log_10_ transformed. Following retesting, the transformed variables were normally distributed and met criteria for parametric statistics. The CRT did not meet assumptions of normality, even following transformation and was therefore recoded into a binary dummy variable. These transformed variables were used in all parametric testing. Age was recoded into six age bands as follows: 18–28, 29–38, 39–48, 49–58, 59–68 and 69–78.

Descriptive statistics are reported in Table 1. A breakdown of the TALE by presence, median age of exposure, and child versus adult exposure is reported in Table 2.

**TABLE 1:** Descriptive statistics.

Variable	Mean (95% confidence intervals)	Std. error mean	Median	SD	Range	Skewness	Kurtosis
Age	34.25 (32.22–36.27)	1.02	32	11.81	48	0.7	−0.28
DUP^a^	13.03 (8.39–17.67)	2.34	1.38	25.02	119.97	0.33	−0.83
SAPS	8.01 (7.23–8.78)	0.39	8	4.45	21	0.13	−0.15
SANS^a^	4.79 (3.9–5.68)	0.45	4	5.12	22	−0.44	−0.72
CDSS^a^	3.94 (2.93–4.95)	0.51	1	5.72	25	0.17	−1.24
CRT^a^	0.17 (0.05–0.28)	0.06	0	0.69	4	4.18	16.5
TALE 14	1.98 (1.67–2.3)	0.16	2	1.82	8	0.88	0.28

	Frequency	Percentage
Sex		
Male	79	59.4
Female	54	40.6
Service disengagement		
No	131	98.5
Yes	2	1.5
Substance use		
Absent	37	27.8
Present	81	60.9
Interpersonal difficulties		
Absent	52	39.1
Present	81	60.9
Work stress		
Absent	102	76.7
Present	31	23.3
Trauma‐exposure TALE 14		
Childhood	63	47.4
Adulthood	34	25.6

^a^ Raw data were used to generate descriptive statistics instead of the log transformed or binary versions of the variables.

**TABLE 2:** A breakdown of TALE items by presence, mean age and child/adult exposure.

TALE item	Absent	Present	Percentage present	Median age of occurrence	Child^a^	Adult^a^
1. Exposure to war	131	2	1.5	—	—	—
2. Loss/permanent separation to someone close to Px	72	61	45.9	30	11	36
3. Period of separation to someone close to Px	83	50	37.6	14	22	17
4. Sudden/unexpected change in circumstances	93	40	30.1	24	9	21
5. Bullying/harassment	110	23	17.3	15	8	4
6. Discrimination	127	6	4.5	31	1	2
7. Someone close to Px humiliating Px	121	12	9	19	2	3
8. Someone close to Px being violent/aggressive	118	15	11.3	20	2	4
9. Witnessing physical violent/verbal aggression in home	120	13	9.8	13	2	1
10. Someone unknown being violent/aggressive	125	8	6	22	2	5
11. Feeling unsafe, unloved or unimportant during childhood	114	19	14.3	8	4	—
12. Going hungry, thirsty, not having clean clothes, safe place during childhood	128	5	3.8	11	2	—
13. Unwanted sexual contact before 16th birthday	124	9	6.8	7	2	—
14. Unwanted sexual contact after 16th birthday	132	1	0.8	30	—	1

^a^ Child and adult ages of trauma may not equal total number of recorded traumas. If no age was indicated in the assessment, an age was not recorded here.

A series of independent samples t‐tests and chi square tests were conducted to investigate sex differences. A small significant difference in SAPS scores between males > females was found (Cohen's *d* = 0.38). A moderate significant difference in CDSS scores between males > females found (Cohen's *d* = 0.5). Males had a small increased likelihood of substance use compared to females (Phi = −0.21).

### 3.3 Exposure to Trauma

The Tale 14 rate of trauma‐exposure was 73.7%, with a median of 2 incidents. Childhood exposure to trauma was reported by 47.4% of participants, and adulthood exposure by 25.6%. Rates of CRT were low, with only 6% reporting CRT, with a mean of 0.17. Interpersonal difficulties were reported by 60.9% of participants and 23.3% of participants reported experiencing work stress (see Table 2).

### 3.4 Association Between Variables

Bivariate correlations were carried out to assess the associations between the variables (see Table 3). SAPS and SANS scores, and DUP length and SANS scores were positively, weakly associated.

**TABLE 3:** Correlation table.

Variable	DUP	SAPS	SANS	CDSS
DUP	—			
SAPS	0.14	—		
SANS	0.36*	0.32*	—	
CDSS	0.12	−0.17	0.22*	—
TALE 14	0.01	0.13	0.16	−0.04

*Note: df* = 114–123.

* *p* < 0.05.

### 3.5 Trauma‐Exposure as Symptom Predictor

A hierarchical multiple regression investigated the ability of interpersonal difficulties, work stress and Total TALE 14 scores to predict SAPS scores, when controlling for sex (see Table 4). Interpersonal difficulties were found to have a significant, independent association with SAPS scores in Model 3 of the Regression.

**TABLE 4:** Hierarchical regressions investigating predictive ability of TALE 14 scores.

Model	Independent variable	*F* change	*df*	*p*	Adjusted *R* ^2^	*β*
Dependent: SAPS, *F* = 2.122, *df* = 5, 123, *p* < 0.067						
1	Sex	4.62	1, 127	0.034*	0.27	−0.166
2	Interpersonal difficulties	0.013	2, 126	0.911	0.020	−0.48
3	Work stress	5.37	3, 125	0.022	0.053	−0.179
4	CRT	0.212	4, 124	0.646*	0.047	−0.045
5	TALE 14	0.39	5, 123	0.533	0.042	0.061
Dependent: SANS, *F* = 3.126, *df* = 5, 108, *p* = 0.011*						
1	Substance use	4.27	1, 112	0.041*	0.028	0.233
2	Work stress	6.28	1, 111	0.014*	0.072	−0.285
3	Interpersonal difficulties	0.939	1, 110	0.335	0.071	0.021
4	CRT	0.84	1, 109	0.362	0.070	−0.07
5	TALE 14	2.91	1, 108	0.091	0.086	0.17
Dependent: CDSS, *F* = 1.194, *df* = 4, 125, *p* = 0.317						
1	Work stress	1.663	1124	0.2	0.005	−0.058
2	Interpersonal difficulties	0.636	1123	0.427	0.002	−0.098
3	CRT	2.187	1, 122	0.142	0.012	−0.141
4	TALE 14	0.300	1, 121	0.585	0.006	0.056
Dependent: DUP, *F* = 0.398, *df* = 3, 110, *p* = 0.755						
1	Age	0.181	1112	0.672	−0.007	0.038
2	CRT	0.006	1, 111	0.938	−0.016	0.003
3	TALE 14	1.008	1, 110	0.318	−0.016	−0.096

* *p* < 0.05.

A hierarchical multiple regression investigated the ability of interpersonal difficulties, work stress, and Total TALE‐14 scores to predict SANS scores, when controlling for substance use (see Table 4). Work stress was found to be significantly and independently associated with SANS scores.

A linear multiple regression was carried out to investigate the ability of interpersonal difficulties, work stress, and Total TALE 14 scores to predict CDSS scores (see Table 4). No statistically significant relationships were found.

A hierarchical multiple regression investigated the ability of interpersonal difficulties, work stress, and TALE scores to predict DUP lengths (see Table 4). The model was statistically significant (*F* (1, 112) = 7.01, *p* = 0.009), with work stress explaining 5.9% of the variance in DUP lengths. Work stress was significantly associated with DUP length (*β* = −0.24, *p* = 0.009).

Exploratory hierarchical linear regression analysis examined whether childhood trauma or adult trauma based on the age of TALE 14 trauma‐exposure predicted clinical symptoms. Analyses did not find any significant association with the dependent variables.

## 4 Discussion

### 4.1 Summary

The current study examined a 6‐year dataset of clinical histories taken as part of intake assessments in an early intervention in psychosis service to identify reported trauma‐exposure, characterise the reported traumas, and examine clinical correlates. The study hypothesised that there would be a low rate of trauma recording, in line with the findings of Neill and Read (2022) and Read et al. (2018).

In contrast to the hypothesis, the current study found that trauma was frequently recorded within case histories. This was most frequently a ‘*permanent/ significant separation from someone close*’ or a ‘*sudden change in circumstances*’. The regression analyses found no significant relationship between reported trauma‐exposure and psychotic symptoms. However, a significant relationship was found between stressful life events (interpersonal stressors) and SAPS scores, and stressful life events (work‐related stress) and SANS scores.

### 4.2 Recorded Rates of Trauma

Contrary to the hypothesis of this study, the results found that trauma was frequently recorded in the assessments. The results indicated that the majority of participants had some exposure to traumatic events (73.7%), which was recorded by clinicians. This finding aligns with other studies reporting trauma‐exposure in psychosis populations (Conus et al. 2007 [83%]; DeTore, Gottlieb, and Mueser 2021 [80%]; Neria et al. 2002 [68.5%]). While positive, there are some notable nuances in the data. The traumas recorded were typically broader stressful or attachment‐related life experiences, rather childhood sexual, physical abuse or neglect. In fact, the documented childhood sexual abuse rate was lower than Irish and international general population samples (McGee et al. 2002; Pereda et al. 2009), and in line with underreporting in previous Irish (Rossiter et al. 2015), and UK psychiatric samples (Neill and Read 2022).

It was noticeable that these data were included as part of detailed clinical assessment, but was not systematically recorded or summarised, nor did it lead to further diagnostic assessment of PTSD or C‐PTSD. Detailed clinical assessments, particularly within specialist services where there is a prominent focus on developmental and early life risk factors, may glean information about trauma‐exposure both deliberately and incidentally. The context of an initial assessment with an individual with a FEP means that by definition the trauma‐exposure will not represent the primary reason for help‐seeking. The absence of a systematic, trauma‐informed assessment protocol increases the risk that an event may be recorded but its relevance and sequalae missed. Longitudinally, the benefits of the trauma‐informed history taking may be lost as the patient progresses through their treatment, if the trauma component of their presentation is not systematically incorporated into the treatment plan. This may be an intermediate link to appropriately deploying standardised assessments for PTSD or C‐PTSD.

Forty‐seven percent of the participants reported childhood trauma‐exposure, indicating the potential importance of adverse early experiences in the development of psychosis. Although the variables of interest are not identical, this finding is in line with DeTore, Gottlieb, and Mueser's (2021) prospective study and Conus et al. (2007) retrospective study of PTSD in FEP populations which indicated that a significant amount of the trauma‐exposure in FEP cohorts occurs before the age of 18. For instance, in DeTore et al., the average age for all trauma‐exposures was 18 or under. The current literature suggests that individuals experiencing psychosis may benefit from trauma therapies, such as TF‐CBT or EMDR (Van den Berg et al. 2015), where difficulties of PTSD or C‐PTSD are identified.

In the current study, and similarly to Conus et al. (2007) and DeTore, Gottlieb, and Mueser (2021), attachment‐oriented items were prominent (i.e., Item 2: Loss/permanent separation from someone close to the patient; Item 3: a period of separation from someone close to the patient). The importance of attachment‐oriented items aligns with a range of recent findings on the role of attachment difficulties as a mediator in the development of psychosis (e.g., Degnan et al. 2022; Grady et al. 2024), as well as the development of novel psychological interventions targeting attachment difficulties in psychosis (Airey, Berry, and Taylor 2023).

Notably, later life experiences such as interpersonal stressors and work‐related stress were related to psychotic symptoms and were commonly reported in participant assessments. The significant relationship between work‐related stress and SANS scores supported research implicating stressful life events and work‐related stress specifically in the development of psychosis (Amin 2023; Collip et al. 2011).

The significant relationship between interpersonal stressors and SAPS scores is consistent with research by Bentall et al. (2014). It also supports the previously identified link between adulthood bullying and positive symptoms (O'Moore et al. 1998).

### 4.3 Trauma and Symptom Severity

The absence of a significant relationship between the retrospective assessment of trauma‐exposure and symptom severity contrasts with the previous literature (Carr, Hardy, and Fornells‐Ambrojo 2018; Larkin and Read 2008; Matheson et al. 2013; Shelvin et al. 2007). Childhood exposure to trauma also had no relationship with symptom severity, again contrasting with previous literature (Bentall et al. 2014; Humphrey et al. 2022). A possible explanation for this finding is that other factors mediate the influence trauma‐exposure has on symptom severity. For example, attachment and cannabis‐use have been shown to be mediating factors in the relationship between childhood trauma and psychosis (Grady et al. n.d.; Humphrey et al. 2022; Kiburi et al. 2021). Finally, methodological issues with retrospective assessment of trauma‐exposure based on case‐histories may also have impacted the findings. This methodology does not have the advantage of being able to explore trauma‐exposure experiences with participants to understand the nuances of the experiences, the impact or trauma‐related symptomatology.

### 4.4 Strengths and Limitations

The study's strengths include the assessment of a complete FEP cohort using validated diagnostic and clinical instruments; the use of a standardised assessment measure ensured a reliable definition of trauma‐exposure reporting. Furthermore, the sample size was appropriately powered for the statistics used. This study design is a partial replication of the study design used by Conus et al. (2007) in a similar FEP cohort in Melbourne, Australia.

Limitations include that the TALE measure was originally designed as a questionnaire or interview tool. This is a novel use of the TALE to retrospectively categorise and classify the information recorded in clinical assessments. Previous studies using this methodology pre‐date the development of the TALE and used other trauma‐exposure frameworks. As a result, the researchers may have incorrectly attributed difficult life events as traumatic. Similarly, only a single reviewer examined the records and determined whether TALE items were recorded or not. This is a significant limitation and is contract to other studies in the area (i.e., Sampson and Read 2017), where two reviewers examined the files independently and came to an agreement on inclusion or not, reflecting how trauma‐exposure can be recorded ambiguously within assessments. Nonetheless, the rates reported in this study are in line with other trauma‐exposure assessed in other FEP cohorts (Conus et al. 2007; DeTore, Gottlieb, and Mueser 2021; Neria et al. 2002). The original assessments were performed by senior clinical staff. However, interviewing participants would allow for a more detailed assessment and classification of trauma. The retrospective use of the TALE should be validated in a further study. This study did not measure trauma severity or PTSD symptoms, which may have a stronger link to symptomology (Conus et al. 2007; DeTore, Gottlieb, and Mueser 2021). It is reasonable to assume that the more severe trauma‐exposure, the more severe their psychosis symptoms may be. In a prospective study using the TALE, recurrent trauma‐exposure but not trauma‐exposure alone was a significant predictor of symptoms severity (Grady et al. n.d.).

## 5 Conclusion

The high rates of recorded trauma‐exposure in the cohort highlighted the ability of services to identify trauma‐exposure through a thorough clinical assessment. This study did not find a significant relationship between trauma‐exposure and symptom severity, which may reflect that the relationship is complex and likely mediated through factors not assessed in this study. Trauma‐exposure should be considered in structured assessments and treatment planning, including pharmacological, social, and psychological interventions, to deliver comprehensive trauma‐informed care.

## Ethics Statement

The research ethics committees at University College Dublin and Saint John of God Community Services CLG approved this study. The study was conducted in line with the Declaration of Helsinki—Ethical Principles for Medical Research Involving Human Subjects (World Medical Association 2022).

## Conflicts of Interest

The authors declare no conflicts of interest.

## Supporting information


**Data S1:** Supporting Information.

## Data Availability

The data that support the findings of this study are not openly available due to reasons of sensitivity based on the research ethics approval for this current retrospective chart review, which also included a data protection impact assessment we do not have permission to share the data publicly. Data are located in controlled access data storage at DETECT, Early Intervention in Psychosis Services, under the auspices of St John of God Community Services.
