# Supplementary material for: Recorded Rates of Trauma‐Exposure in a Retrospective Epidemiologically Complete First‐Episode Psychosis Cohort
Source: Early Interv Psychiatry. 2024 Aug 31;19(1):e13610. doi: 10.1111/eip.13610 (PMC11729992; doi:10.1111/eip.13610)
Supplement: Supplementary file 1 — Data S1: Supporting Information. [file EIP-19-0-s001.docx]

**Supplemental. Table 1**

*Testing sex, age substance differences on clinical variables*

| Variable | T-value | df | Variable | F-value | df |
| --- | --- | --- | --- | --- | --- |
| *Sex* |  |  | *Age* |  |  |
| TALE | .06 | 131 | TALE | 1.07 | 4 |
| TALE 14 | -.66 | 131 | TALE 14 | 1.26 | 4 |
| SAPS | 2.1* | 127 | SAPS | .99 | 4 |
| SANS | .15 | 92 | SANS | 2.16 | 4 |
| CDSS | -2.13 | 79 | CDSS | .73 | 4 |
| DUP | 1.84 | 112 | DUP | .48 | 4 |
| *Substance use* |  |  |  |  |  |
| TALE | -.49 | 116 |  |  |  |
| TALE 14 | -.23 | 116 |  |  |  |
| SAPS | -.49 | 112 |  |  |  |
| SANS | 2.21* | 85 |  |  |  |
| CDSS | .02 | 31.24 |  |  |  |
| DUP | 1.12 | 99 |  |  |  |
| *Chi square test of independence* | | | Pearson chi-square | | df |
| Substance use and interpersonal difficulties | | | .13 | | 1 |
| Substance use and work stress | | | 2.68 | | 1 |
| Substance use and child/adult TALE exposure | | | .02 | | 1 |
| Substance use and child/adult TALE 14 exposure | | | .86 | | 1 |
| Substance use and CRT | | | 1.39 | | 1 |
| Sex and interpersonal difficulties | | | .1 | | 1 |
| Sex and work stress | | | 2.03 | | 1 |
| Sex and child/adult TALE exposure | | | 1.38 | | 1 |
| Sex and child/adult TALE 14 exposure | | | 3.05 | | 1 |
| Sex and CRT | | | 7.76** | | 1 |
| Age and interpersonal difficulties | | | 5.53 | | 4 |
| Age and work stress | | | 14.57** | | 4 |
| Age and CRT | | | 9.64* | | 4 |

*Note.* *** = p < .001, ** = p < .01, * = p < .05
